# A Nurse-Led Telehealth Program for Diabetes Foot Care: Feasibility and Usability Study

**DOI:** 10.2196/40000

**Published:** 2023-06-06

**Authors:** Hsiao-Hui Ju, Rashmi Momin, Stanley Cron, Jed Jularbal, Jeffery Alford, Constance Johnson

**Affiliations:** 1 The University of Texas Health Science Center at Houston Cizik School of Nursing Houston, TX United States; 2 Affiliates of Family Medicine Spring, TX United States; 3 Sweetwater Medical Associates Sugar Land, TX United States

**Keywords:** diabetes mellitus, type 2, telehealth, telemedicine, foot care education, self-management, self-care, preventive health services, patient education, diabetes complications, diabetic foot

## Abstract

**Background:**

Diabetes mellitus can lead to severe and debilitating foot complications, such as infections, ulcerations, and amputations. Despite substantial progress in diabetes care, foot disease remains a major challenge in managing this chronic condition that causes serious health complications worldwide.

**Objective:**

The primary aim of this study was to examine the feasibility and usability of a telehealth program focused on preventive diabetes foot care. A secondary aim was to descriptively measure self-reported changes in diabetes knowledge, self-care, and foot care behaviors before and after participating in the program.

**Methods:**

The study used a single-arm, pre-post design in 2 large family medical practice clinics in Texas. Participants met individually with the nurse practitioner once a month for 3 months using synchronous telehealth videoconferencing. Each participant received diabetes foot education guided by the Integrated Theory of Health Behavior Change. Feasibility was measured with rates of enrollment and program and assessment completion. Usability was measured with the Telehealth Usability Questionnaire. Diabetes knowledge, self-care, and foot care behaviors were measured with validated survey instruments at baseline, 1.5 months, and 3 months.

**Results:**

Of 50 eligible individuals, 39 (78%) enrolled; 34 of 39 (87%) completed the first videoconference and 29 of 39 (74%) completed the second and third videoconferences. Of the 39 who consented, 37 (95%) completed the baseline assessment; 50% (17/34) of those who attended the first videoconference completed the assessment at 1.5 months, and 100% (29/29) of those who attended the subsequent videoconferences completed the final assessment. Overall, participants reported a positive attitude toward the use of telehealth, with a mean Telehealth Usability Questionnaire score of 6.24 (SD 0.98) on a 7-point scale. Diabetes knowledge increased by a mean of 15.82 (SD 16.69) points of 100 (*P*<.001) from baseline to 3 months. The values for the Summary of Diabetes Self-Care Activities measure demonstrated better self-care, with participants performing foot care on average 1.74 (SD 2.04) more days per week (*P*<.001), adhering to healthy eating habits on average 1.57 (SD 2.12) more days per week (*P*<.001), and being physically active on average 1.24 (SD 2.21) more days per week (*P*=.005). Participants also reported an improvement in the frequency of foot self-examinations and general foot care behaviors. The mean scores for foot care increased by a mean of 7.65 (SD 7.04) points (scale of 7 to 35) from baseline to 3 months postintervention (*P*<.001).

**Conclusions:**

This study demonstrates that a nurse-led telehealth educational program centered on diabetes foot care is feasible, acceptable, and has the potential to improve diabetes knowledge and self-care, which are precursors to preventing debilitating foot complications.

## Introduction

### Background

The social distancing and isolation associated with the COVID-19 pandemic have increased amputation risk among patients with diabetes mellitus, owing to the drastic alterations in diabetes care and interruptions in diabetes patient education programs [1,2]. Diabetes can lead to severe and debilitating foot complications, such as infections, ulcerations, and amputations. Despite substantial progress in diabetes care, diabetic foot ulceration remains a major challenge in managing this chronic condition that is associated with high morbidity and mortality rates [1-3]. For example, the 5-year mortality rate in individuals with diabetic foot ulcers is >50%, which is similar to or worse than that in individuals with common cancers [2]. Worldwide, 40 to 60 million people with diabetes experience foot and lower limb complications, and 75% of lower extremity amputations are performed in patients with diabetes [4,5]. From 2009 to 2015, data on nontraumatic lower extremity amputation (NLEA) procedures showed a 62% increase in minor amputations and a 29% increase in major NLEAs [6]. After lower extremity amputation from diabetic foot ulcer, the 3-year mortality rate can reach 70% [7]. The significant increase in the prevalence of NLEA underscores the need to implement effective methods to improve patient outcomes within the primary care setting, where diabetic foot disease is diagnosed, treated, and managed. Studies have shown that deficiencies in early preventive diabetic foot care, such as a lack of patient education and provider management, may contribute to increased amputation rates [8,9]. Although the American Diabetes Association recommends regular foot exams [10], only 30%-33% of provider-patient visits adhere to this guideline [11,12], leading to missed opportunities to address foot care in this population. Despite the prevalence of diabetes-related foot complications, foot care education and prevention programs for people with diabetes are lacking [4].

Early detection and reporting of diabetic foot problems are critical to preventing devastating outcomes and invasive treatment, thus improving quality of life and reducing mortality rates [13]. However, people with diabetes may be unaware of their foot risks, and in-office foot examinations are often overlooked [11,12,14-16]. Without adequate instruction, patients are not equipped with the necessary self-care skills to effectively care for this chronic condition and delay foot care until problems develop, at which time invasive treatment may be necessary. During the COVID-19 pandemic, diabetes-related amputation rates have increased globally [17,18]. Therefore, there is an urgent need to engage patients in the early detection of foot problems, improve their understanding of foot risks, and avoid treatment delays. A major health care innovation during the pandemic is the widespread adoption of telehealth technology. The upsurge in telehealth services during the pandemic provides new communication tools for collaboration between patients and health care professionals [19]. Through mobile devices and internet networks, telehealth includes electronic technology and remote sharing of health information that can be used to prevent diabetes and its complications [20].

Technology continues to revolutionize communication between patients and clinicians via telehealth. The use of telehealth provides an avenue for clinician and patient collaboration between rural and urban clinic sites for the purpose of managing advanced diabetic foot disease [21]. Before videoconferencing technologies were available, clinicians relied on digital cameras, fax machines, desktop computers, modems, phone conversations, and emails to assess ulcer size and infection status [21]. Advancements in high-speed internet and video technology allow health care providers to monitor patients’ foot ulcerations and provide guidance on ulcer healing remotely [22]. In terms of ulcer healing and recovery time, telehealth care using web-based consultation and digital imaging has been shown to be comparable with usual treatment [22]. Although recent literature has explored the potential value of telehealth in achieving glycemic control and monitoring existing diabetes complications, research on telehealth technology for foot care education and prevention is lacking. Previous studies have focused on patients with advanced foot ulcerations and chronic nonhealing foot wounds [22]. However, knowing that patients with diabetes may have a lower level of sensual perception and trouble healing [22], preventive foot care education based on individual risks is crucial to avoid serious foot injuries. Therefore, we implemented a theory-based, patient-centered telehealth program delivered using synchronous videoconferencing technology as a potential strategy to prevent diabetes-related foot complications.

### Objectives

The primary aim was to examine the feasibility (enrollment, program, and assessment completion) and usability of a telehealth foot care educational program focused on preventive foot care measures. Specifically, the primary objectives were to assess (1) the demand for intervention by people with type 2 diabetes (whether people enroll in the program to learn about foot care) [23], (2) the completion of the telehealth educational program (whether people participate in the videoconferences and complete the surveys) [23], and (3) the usability of the telehealth educational program for people with type 2 diabetes (whether people find the educational program satisfying and want to continue using telehealth) [23]. Usability was measured using the Telehealth Usability Questionnaire (TUQ) [24]. The secondary aim was to descriptively measure self-reported changes in diabetes knowledge, self-care, and foot care behaviors before and after participating in the program.

### Theoretical Framework

The Integrated Theory of Health Behavior Change (ITHBC) [25] guided this study [25]. The theory posits that a person’s knowledge, beliefs, and social facilitation affect their self-care and ultimately lead to the adoption of self-care behavior [25]. Knowledge of diabetes was measured using the Diabetes Knowledge Scale (DKS) [26]. Social facilitation, defined as the quality of positive social interactions, was measured using the TUQ [24]. Together, these factors influence a person’s self-care, defined as the ability to apply new knowledge to maintain health, which was measured by the Summary of Diabetes Self-Care Activities (SDSCA) [27]. Finally, the factors leading to the adoption of behavior change, which is to incorporate foot self-care practices into one’s daily routine, were measured using the Diabetes Foot Self-Care Behavior Scale (DFSBS) [28].

## Methods

### Overview

We used a one-arm, pre-post design to assess the feasibility and usability of the telehealth diabetes program. Adults with type 2 diabetes recruited from 2 large family medical practice clinics in Texas completed surveys at baseline, at 1.5 months, and at 3 months after the telehealth diabetes program intervention.

### Ethics Approval

This study was reviewed and approved by the University of Texas Health Science Center Institutional Review Board (HSC-SN-21-0240).

### Recruitment

The principal investigator provided study flyers to 2 large family medical practice clinics that provide primary care services to patients of all ages living in the urban Houston–The Woodlands–Sugar Land metropolitan area. The institutional review board–approved study flyers were posted in the clinics’ waiting areas. The medical practitioners (physicians and nurse practitioners) at each clinic recruited participants. Interested patients then contacted the principal investigator via phone or SMS text message to determine eligibility for participation. Participants were eligible if they (1) were aged 18 to 64 years, (2) had access to the internet with a smartphone or a computer, (3) had a history of type 2 diabetes, and (4) were able to speak and read English. Patients were excluded if they had (1) a history of leg or foot ulcers or amputations or (2) a diagnosis of gestational diabetes. If the patient was deemed eligible, the principal investigator explained the study and obtained informed consent in person or electronically via Research Electronic Data Capture (REDCap; Vanderbilt University for Management of Research Data) [29,30] through a secure link sent to the participant’s email.

### Intervention

The principal investigator scheduled three 1-hour monthly interactive Zoom (Zoom Video Communications) videoconferences with each participant to discuss comprehensive diabetes foot care and self-care behaviors. Each telehealth session followed a carefully curated outline based on the ITHBC [25] and was planned by an interdisciplinary team with expertise in diabetes, nursing education, and health care technology or informatics. Sources of the educational materials and recommendations were obtained from the American Diabetes Association [31,32], the International Diabetes Federation [4], and the Health Resources and Services Administration, an agency of the US Department of Health and Human Services [31,33]. Sessions were facilitated by the principal investigator, a family nurse practitioner with additional board certification in nursing education. During the first telehealth visit, participants were educated about foot care practices, including foot inspection, toenail care, foot cleaning, and appropriate footwear and the rationales for these behaviors. The second telehealth session focused on maintaining healthy eating habits and adhering to the recommended course of action with the support of family and providers. During the last telehealth visit, the principal investigator reviewed key concepts of diabetes foot care with participants, supported self-goal planning and monitoring, and talked about office examinations for sensory neuropathy and peripheral vascular disease. Additionally, the principal investigator discussed each question on the Health Resources and Services Administration Foot Care Quiz from the American Diabetes Association [31] with the participant and went over any incorrect responses to make sure the participant understood the rationales. The learning objectives of the telehealth educational program were to (1) identify the signs and symptoms of foot ulcerations, (2) describe foot care and diabetes self-care behaviors, and (3) discuss proper follow-up for foot care.

### Study Procedure

Once participants gave consent, the REDCap [29,30] link automatically directed them to subsequent pages, where we obtained demographic information and the baseline survey data. At baseline, participants were asked to complete (1) a demographics questionnaire, (2) the DFSBS [28] to assess baseline foot care behaviors, (3) the DKS [26] to measure baseline knowledge of diabetes, and (4) the SDSCA [27] to assess participants’ self-care. At 1.5 months after the first telehealth visit, we invited participants to complete midintervention surveys electronically via REDCap [29,30]. The midintervention surveys included (1) the DFSBS [28], (2) the DKS [26], and (3) the SDSCA [27]. Three months after the first telehealth visit, we invited participants who attended all 3 telehealth visits to complete postintervention surveys electronically via REDCap [29,30]. The postintervention assessments included (1) the DFSBS [28], (2) the DKS [26], (3) the SDSCA [27], and (4) the TUQ [24] to provide information on the usability of the telehealth program. In the end, participants received a US $50 gift card sent to their email address for completing the study.

### Measurement

To meet the primary aim of the study, we measured the feasibility and usability of the telehealth program. At baseline, participants were asked to complete a demographics survey including age, gender, race and ethnicity, level of education, marital status, employment status, and diabetes duration. To assess feasibility, we measured the rates of enrollment, retention, and assessment (ie, survey) completion. The enrollment rate was the proportion of those meeting the inclusion criteria who enrolled, while the retention rate was the proportion who enrolled and completed the study. The assessment completion rate was calculated as the proportion of assigned surveys at each time point that were completed.

The usability of the program was measured by participants’ perceived telehealth usability using the TUQ [24]. The TUQ provided information on the social facilitation construct of the ITHBC model [25] to measure the acceptability, usability, and satisfaction of the participants’ telehealth experience [24]. The 21 questions within the TUQ are divided into 5 subcategories: usefulness, ease of use, effectiveness, reliability, and satisfaction. All subcategories of TUQ demonstrated a Cronbach coefficient α of .81 to .93, indicating good to excellent internal consistency reliability [24]. Evidence of content validity has also been reported [24]. The 21-item questionnaire asked participants to rate their telehealth experience on a 7-point Likert scale (1=strongly disagree and 7=strongly agree) [24,34].

To meet the secondary aim of investigating the preliminary effects of participation in the telehealth educational program, we gathered self-reported changes in diabetes knowledge, self-care, and foot care behaviors before and after program implementation. The DKS [26] was used to assess participants’ knowledge of diabetes. The scale consists of 18 true or false questions on knowledge of nutrition, exercise, foot health, and overall diabetes monitoring with 2 more specific questions for people taking insulin [26]. Both the general DKS and the insulin subscale demonstrated reliability with a Cronbach coefficient α of .77 and .84, respectively [35]. The scale has demonstrated validity [35]. The questions were scored from 0 to 100, where higher scores represented better diabetes understanding. The average was obtained by adding all the scores and dividing by the number of people who completed the survey.

The participants’ self-care in managing diabetes was measured by the SDSCA [27]. The interitem and test-retest reliability demonstrated high to moderate correlations of .47 and .40, respectively [27]. The 13-item questionnaire is divided into sections of diet, exercise, blood glucose testing, foot care, and smoking status, with 2 additional questions for cigarette smokers, and has shown evidence of validity [27]. In the questionnaire, participants were asked to rate the number of days (0-7) they performed a specific self-care activity in the past 7 days. Scores were calculated by obtaining the mean number of days for each section [27]. For the cigarette smoking status, if the respondent was a cigarette smoker, 1 point was entered and added to the number of cigarettes smoked per day [27].

The DFSBS [28] was used to measure the participants’ foot self-care behaviors [28]. The DFSBS has a Cronbach coefficient α of .73, and the intraclass correlation coefficient over 2 weeks was 0.92 (*P*<.001); both coefficients indicate that the DFSBS scale is reliable as a screening tool for daily foot care activities [28]. The scale has shown evidence of validity [28]. The 7-item scale is divided into 2 parts. The first section asked participants to rate the number of days they performed foot care in the past 7 days (0 days, 1-2 days, 3-4 days, 5-6 days, or 7 days). The second part asked them to rate the frequency they performed general foot activities (categorized into never=1, rarely, sometimes, often, or always=5). The items in both sections were added, with a range of 7-35, where higher scores indicated better foot self-care [28,36].

### Statistical Analysis

Statistical analysis was conducted using SAS software for Windows (version 9.4; SAS Institute Inc) [37]. We calculated descriptive statistics to assess the feasibility, usability, and self-reported changes in diabetes knowledge, self-care, and foot care measures before and after participating in the telehealth educational program. Paired-sample *t* tests (2-sided) were conducted to determine if there were any significant differences between the preintervention and 3-month postintervention scores.

## Results

### Sample Characteristics

Of the 39 participants who consented to participate in the pilot study, 4 did not take part in the subsequent videoconferences because of schedule conflicts. Another participant tried to join the initial videoconference on the wrong date. A total of 29 participants completed all videoconferences at 3 months. Most of the dropouts had attended some college, were married, were employed full-time, were aged 50-59 years, and had diabetes for 1-5 years. They did not differ from the participants who completed the study. Table 1 presents the characteristics of the study sample. The participants were predominately employed full-time (27/39, 69%), male (24/39, 62%), married or cohabiting (29/39, 74%), and aged 50-59 years (20/39, 51%). The level of education varied, with 11 (28%) having completed a bachelor degree, 7 (18%) having a master or doctoral degree, and 5 (13%) having high school or less than high school education. The sample was diverse: 16 (41%) were White, 9 (23%) were African American, 9 (23%) were Hispanic, and 4 (10%) were Asian.

**Table 1:** Sample characteristics of participants (N=39).

Characteristics		Participants, n (%)
**Age range (years)**		
	30-39	7 (18)
	40-49	5 (13)
	50-59	20 (51)
	60+	6 (15)
	Missing	1 (3)
**Gender**		
	Male	24 (62)
	Female	14 (36)
	Missing	1 (2)
**Marital status**		
	Single	5 (13)
	Married or cohabiting	29 (74)
	Divorced	4 (10)
	Missing	1 (3)
**Employment status**		
	Unemployed	7 (18)
	Part-time	2 (5)
	Full-time	27 (69)
	Retired	2 (5)
	Missing	1 (3)
**Educational attainment**		
	Less than high school	3 (8)
	High school	2 (5)
	Some college	12 (30)
	Associate degree	3 (8)
	Bachelor’s degree	11 (28)
	Master’s degree	4 (10)
	Doctorate	3 (8)
	Missing	1 (3)
**Race and ethnicity**		
	White	16 (41)
	African American	9 (23)
	Hispanic	9 (23)
	Asian	4 (10)
	Missing	1 (3)
**Diabetes duration (years)**		
	Less than 1	8 (20)
	1-5	14 (36)
	6-10	10 (26)
	More than 10	6 (15)
	Missing	1 (3)

### Feasibility: Enrollment, Retention, and Assessment Rates

Of the 50 individuals who were eligible for the study, 39 (78%) consented to participate. After consenting and before the first videoconference, 5 participants (5/39, 13%) dropped out of the study. The availability of participants and the time commitment were the most common issues, as 1 participant shared that he was offered a new job after he consented and could no longer find the time for the study. One participant had other, more critical health issues that needed to be resolved. Of the 39 who consented, 34 (87%) completed the first videoconference and 29 (74%) completed the second and third videoconferences. Therefore, 74% (29/39) finished the study, defined as attending all 3 videoconferences. The percentage of participants completing the baseline assessment was 95% (37/39). The percentage of participants completing assessments at 1.5 months was 50% (17/34). The percentage of participants completing final assessments at 3 months was 100% (29/29). On average, the assessment completion rate for all surveys was approximately 82%.

During the abrupt outbreak of COVID-19, many companies switched to virtual technological platforms such as Zoom to meet. All but 2 of the participants in the study were familiar with Zoom technology. One participant initially had challenges using the technology and needed his daughter to help with joining Zoom meetings. However, by the third videoconference, he successfully connected to Zoom by himself. Another participant had difficulty allowing the camera to turn on in the Zoom application using his mobile device. After getting help from the principal investigator, he was also able to connect with audiovisual media using his smartphone. Most participants used their cellular phones to connect, likely owing to the easy and convenient nature of mobile technology. However, while those who lived in the urban area had no issues with connectivity, 2 participants in the rural areas had poor connection quality and speed, causing the virtual conferences to be rescheduled.

One participant logged in a day early and never rejoined because of a schedule conflict. Several participants also had to reschedule the videoconferences due to work schedule conflicts, severe weather, or simply forgetting, despite calendar reminders sent to their emails. Technology also relies on electrical power and network signals. On September 14, 2021, a tropical storm swept through Texas, resulting in power outages for many individuals, thereby precluding videoconferences at the originally scheduled time.

### Telehealth Usability

Overall, participants reported a positive attitude toward the use of telehealth in this study with an overall mean TUQ score [24] of 6.24 (SD 0.98) on a scale of 1 to 7 (Table 2). They found the telehealth modality to be easy to use, effective, useful, and reliable. Participants were satisfied with its overall use and would use it again. The TUQ [24] demonstrated a Cronbach α of .97, indicating excellent reliability.

**Table 2:** Summary of participants’ perceptions of telehealth usability (n=29).

Telehealth usability (score scale 1-7)	Score, mean (SD)
Usefulness	6.14 (0.97)
Ease of use	6.50 (0.93)
Effectiveness	6.32 (0.96)
Reliability	5.58 (1.48)
Satisfaction	6.15 (1.18)
Total score	6.24 (0.98)

### Changes in Diabetes Knowledge, Self-care, and Foot Care Measures

The mean scores for diabetes knowledge, self-care, and foot care activities for all participants who took the surveys are presented in Table 3. The paired-sample *t* test analysis revealed that participants’ diabetes knowledge, self-care abilities, and foot care activities were higher at 3 months postintervention than at baseline (Table 4). Diabetes knowledge increased by a mean of 15.82 (SD 16.69) points of 100 (*P*<.001) from baseline to 3 months. Postintervention surveys demonstrated significantly better self-care, with participants testing their blood glucose on average 2.26 (SD 2.47) more days per week (*P*<.001), performing foot care an average of 1.74 (SD 2.04) more days per week (*P*<.001), adhering to healthy eating habits on average 1.57 (SD 2.12) more days per week (*P*<.001), and being physically active on average 1.24 (SD 2.21) more days per week (*P*=.005). Cronbach α for the self-care measure [27] was .82, indicating good internal consistency reliability. Participants also reported an improvement in the frequency of foot self-examinations and general foot care behaviors. The mean scores for foot care increased by a mean of 7.65 (SD 7.04) points (scale of 7 to 35) from baseline to 3 months postintervention (*P*<.001). Cronbach α for the DFSBS [28] was .80, indicating good reliability of the scale.

**Table 3:** Descriptive analysis of changes in foot care behaviors, self-care, and diabetes knowledge.

Variables		Baseline (n=37), mean (SD)	1.5 months (n=17), mean (SD)	3 months (n=29), mean (SD)
Diabetes Foot Self-Care Behavior Scale		17.75 (7.42)	21.59 (7.21)	25.46 (6.20)
**Summary of Diabetes Self-Care Activities (days per week)**				
	General diet	3.34 (1.97)	3.74 (1.45)	4.74 (1.62)
	Blood glucose testing	2.26 (2.34)	2.47 (2.17)	4.28 (2.66)
	Foot care	2.69 (2.25)	3.38 (2.24)	4.40 (2.23)
	Exercise	2.03 (1.70)	2.32 (1.40)	3.16 (1.81)
Diabetes Knowledge Scale		70.48 (17.86)	75.92 (15.81)	83.89 (10.70)

**Table 4:** Paired-sample t test analysis of foot care behaviors, self-care, and diabetes knowledge at baseline and 3 months postintervention.

Variables			Participants, n		Baseline, mean (SD)		Three months, mean (SD)		Difference, mean (SD)		95% CI		*t* (*df*)		Significance (2-tailed), *P* value
Diabetes Foot Self-Care Behavior Scale			26		17.81 (6.75)		25.46 (6.21)		7.65 (7.04)		4.81-10.50		5.54 (25)		<.001
**Summary of Diabetes Self-Care Activities**															
	General diet	29		3.17 (1.96)		4.74 (1.62)		1.57 (2.12)		0.76-2.37		3.99 (28)		<.001	
	BG testing	29		2.02 (2.37)		4.28 (2.66)		2.26 (2.47)		1.32-3.20		4.93 (28)		<.001	
	Foot care	29		2.66 (2.25)		4.40 (2.23)		1.74 (2.04)		0.96-2.51		4.59 (28)		<.001	
	Exercise	29		1.91 (1.71)		3.16 (1.81)		1.24 (2.21)		0.40-2.08		3.02 (28)		.005	
Diabetes Knowledge Scale			21		68.07 (18.81)		83.89 (10.70)		15.82 (16.69)		8.22-23.42		4.34 (20)		<.001

## Discussion

### Principal Findings

This study examined the feasibility, usability, and self-reported changes in diabetes knowledge, self-care, and foot care measures before and after participation in a telehealth educational program that focused on foot care during the COVID-19 pandemic. To our knowledge, this study is the first to assess the feasibility, usability, and self-reported changes in foot care measures of a theory-based synchronous telehealth educational program focused on preventive foot care for patients with type 2 diabetes in primary care. We found that the telehealth program was feasible, easy to use, and acceptable for our participants. Participants reported they were satisfied with the overall use of telehealth technology and agreed they would use telehealth sessions again. In addition, the synchronous telehealth program improved self-reported diabetes knowledge, frequency of foot care, and self-care of diabetes management behaviors. Telehealth has frequently been used to diagnose, evaluate, and treat patients [38], and this study demonstrated that telehealth videoconferences can offer theory-based education on foot care for people with type 2 diabetes.

### Feasibility of the Foot Care Telehealth Program

In terms of feasibility measures, the overall telehealth enrollment, participation, and assessment completion rates were favorable. The videoconferences can be conducted at home or work via mobile phones or computers and do not require traveling, which may be more convenient for many participants and may make patients more willing to participate in the study. Conversations with individuals during the recruitment process revealed that a lack of time, scheduling conflicts, and other health priorities were the most frequent barriers to participation in the study. A possible solution would be to use a website or a phone app that would automatically send text reminders of an upcoming videoconference. If they are unable to attend, participants could then use the website or mobile app to reschedule the videoconference by choosing a time and date that work best for them.

Five participants (5/39, 13%) attended the first videoconference but did not complete the subsequent ones, so we could not confirm changes in their diabetes knowledge, foot care measures, or self-care behaviors. The reasons for not completing the subsequent videoconferences may have been related to participants thinking that they already knew the information presented, a lack of time, and illness. Of note, everyone who participated in the second session also completed the third videoconference. Those who remained after the first telehealth session were interested in learning more about diabetes foot care, while others who lost interest dropped out. The 26% (10/39) rate of dropout is comparable to what has been reported in research evaluating the impact of telehealth programs in patients with type 2 diabetes [39]. The 74% (29/39) completion rate over 3 months resembles what was observed in another study evaluating telehealth to set goals for patients with diabetes [40].

The assessment completion rates were favorable. Compared to the baseline and 3 months, the assessment completion rate was lower at 1.5 months. This may be because many were dealing with the severe weather and power outage as well as additional stressors associated with a COVID-19 surge. Our overall assessment completion rates were high compared with those reported in a previous study that administered telehealth surveys to people with diabetes [38]. Establishing a trusting relationship with participants and offering incentives after each assessment is completed may further improve survey response rates. Sending SMS text message reminders to people who have not finished the assessment surveys may also increase completion rates.

### Telehealth Usability

In terms of usability, participants of various ages and racial and ethnic backgrounds reported that the telehealth technology with videoconferencing was easy to use, useful, and effective. Although those who dropped out of the study did not complete the TUQ, the characteristics of those who responded were similar to those of the dropouts. All participants were able to videoconference with the principal investigator using either their smartphones, desktop computers, or laptops. A few people experienced minor audio and video issues, but these were promptly resolved. These findings are consistent with other research evaluating telehealth interventions in people with diabetes [40]. Scores on the telehealth reliability subsection of the TUQ were lowest, most likely attributable to the severe weather that resulted in a power outage in the Houston area and interrupted internet access, forcing telehealth sessions to be rescheduled. Overall, the telehealth technology was well-liked by the participants, and they reported they would use it again. Although most participants did not have major technical difficulties, individuals who were unfamiliar with the application might find it helpful to have a trial session with the research team before the first scheduled videoconference.

Participants also reported that the videoconferences with the nurse practitioner kept them accountable and helped them learn new information, especially when they could ask questions during the videoconferences and obtain immediate answers. Similarly, in a recent review, videoconferencing telehealth consultation was associated with greater engagement and psychological buy-in, compared with phone consultations, allowing opportunities for social support and real-time discussions [41]. In addition, several participants reported that the telehealth discussions about self-care were therapeutic, informative, and supportive. They noted that the nurse practitioner’s sincere demeanor and compassionate presence when discussing foot care had a favorable effect on their mental well-being while fostering awareness of diabetes foot management. Participants also expressed satisfaction with the quality and practicality of the telehealth-delivered foot care sessions. A drawback of the telehealth videoconferencing modality mentioned by some participants was the absence of the “human touch” one would often have with a clinician during an in-person consultation. These sentiments are similar to those reported in a previous study examining the advantages and disadvantages of telehealth technology in diabetes education [42]. The benefits of telehealth technology still outweigh the limitations, as virtual video platforms were accepted among participants of various ages in our study.

### Changes in Diabetes Knowledge, Self-care, and Foot Care Measures

In terms of the secondary aims, most participants were unaware of the importance and necessity of foot care for people with diabetes or how diabetes affects the feet. The lack of foot care knowledge and inconsistent practice of foot management are also consistent with prior studies [43,44]. After the 3 telehealth videoconferences, participants’ self-reported diabetes knowledge, self-care behaviors, and foot care measures improved significantly. Participants were particularly interested in learning about measures to prevent foot ulcers and injuries (eg, avoiding direct heat sources to the feet, protecting against cold exposure, and using well-fitted shoes and socks) as well as specific foot self-care behaviors (specifically what to look for when examining, moisturizing dry skin, and thoroughly drying the toes and feet) to prevent skin infections and breakdown. Participants were unaware of the need to check the inside of the shoes before putting them on or that moisturizer should not be applied between the toes. Several people reported the difficulty of wearing proper shoes when the temperatures are extremely high in Texas; hence, they chose to go barefoot or wear sandals rather than shoes. Others reported they walk around the house barefoot due to cultural customs. Taking culturally appropriate foot care practices into account could help meet the social and cultural needs of a diverse population. The positive self-reported foot self-care behaviors align with previous studies showing that video-based educational programs are helpful in setting diabetes self-care goals [40], reaching glycemic control, reducing hemoglobin A_1c_ levels [45-48], and decreasing hyperglycemic complications [49].

Participants with various diabetes durations reportedly incorporated foot care activities after the telehealth sessions, suggesting that people with diabetes may overlook or have a limited understanding of diabetes foot care, even if they have had diabetes for years. In fact, several participants said they wished they had the foot care education earlier. According to a recent study [18], most people with diabetes referred to a multidisciplinary foot team did not know the reasons for the referral and were less aware of their risks for foot complications. Our study is novel, as we focused on using telehealth technology to engage patients in diabetes foot care, an area often missed in the primary care setting [15]. Thus, telehealth sessions focusing on diabetes foot care have the potential to ease the profound morbidity and mortality associated with diabetic foot complications, especially during the COVID-19 pandemic. Because of the favorable results from this study, a nurse-led telehealth educational program may be a promising strategy for increasing patients’ awareness of foot care and preventing foot complications.

Our positive results for self-reported changes in knowledge, self-care, and foot care behaviors validate the logic of the ITHBC [25], which we used to inform our intervention design. According to the theory, diabetes knowledge and social facilitation are precursors to engaging in self-care actions, including adhering to foot care, nutrition, and physical activity recommendations. In the telehealth intervention, we offered general diabetes education with a focus on foot care, highlighting the need for self-examination and how to effectively manage diabetes. Participants valued the opportunity to talk about their personal stories and reported that the support offered during the telehealth sessions helped them adhere to practice recommendations. The participants then applied the new knowledge to cultivate self-care behaviors and involvement in foot care practices. As a result of increased diabetes knowledge and supportive social interactions, participants developed skills to plan, monitor, implement, and assess their diabetes self-care. They reported an increase in the number of days per week they adhered to foot care, healthy nutrition, and physical activity recommendations. The theory posits that the commitment to implementing these changes is the short-term result, and the changes in health outcomes are the long-term result. Using this theory, we emphasized patient-centered care by meeting with each participant individually to integrate foot care knowledge and promote positive social facilitation to allow each participant to be more attuned to foot care behaviors and to optimize health outcomes. Patient-centered care recognizes that each participant is unique and yields increased patient satisfaction and improved outcomes [50]. Supported and satisfied participants may be more likely to adhere to treatment regimens, leading to better behavioral and clinical outcomes [50].

### Limitations

Although our study included a diverse population, these promising results from 2 family medical practice clinics in Texas need to be validated in additional settings using a randomized trial study design to determine efficacy and examine long-term clinical outcomes. The feasibility of our diabetes telehealth program may be due to our participants’ easy access to broadband mobile technology and the novelty of attending virtual Zoom sessions, rather than interest in diabetes foot care. With the wide availability of mobile broadband networks in urban areas, most participants connected using their cell phones rather than computers. People in rural settings may experience more challenges accessing broadband connectivity for telehealth services and have decreased access to critically needed health care services [51]. In addition, well-educated patients may be more likely to have extra time and technology resources to participate in telehealth programs. Efforts to improve broadband internet access in all geographical areas are needed to meet the needs of rural communities for remote telehealth services and to improve health outcomes [51]. When developing a telehealth program, we must consider the unique challenges patients may face with broadband mobile technology, including a lack of comfort with using new mobile apps, reduced broadband connectivity in rural areas, lack of resources, and privacy concerns. Participants’ educational levels should also be considered.

We measured patient self-reported outcomes over 3 months and cannot confirm whether the increased foot care behaviors would be sustainable over longer periods of time and would be correlated with decreased incidence of infections and amputations. The scheduling of the monthly telehealth videoconferences varied slightly, despite our effort to maintain consistency, to account for adverse weather conditions, illness, and participants’ various schedules. The schedule variations prove the flexibility of telehealth programs and are a realistic depiction of a practical study. Nurse practitioners should screen patients at risk for diabetic foot disease and advocate for increased access to telehealth technologies for foot care education and support. Ongoing diabetes foot care education and support for all patients are essential to improving diabetes knowledge and self-care behaviors that are precursors to preventing debilitating foot complications.

### Conclusions

Our preliminary results demonstrate that a synchronous telehealth educational program focused on foot care is feasible, easy to use, and acceptable in patients with type 2 diabetes. Our study showed significant improvements in self-reported diabetes knowledge, self-care, and foot practices after 3 months of participation in the program. Telehealth technology is an essential tool for ensuring accessible health care [41]. It represents an innovative path to support patients with diabetes in performing foot care. Using the ITHBC [25], we demonstrated that a telehealth educational program centered on diabetes foot care during the COVID-19 pandemic has the potential to engage patients to be actively involved in managing their health and improve their diabetes knowledge. Therefore, an educational program incorporating telehealth videoconferences to improve diabetes foot care is urgently needed for providing optimal care to people with diabetes. Further investigation is needed to determine the long-term efficacy of a telehealth program to promote diabetes foot care and self-care behaviors.

## Abbreviations

DFSBS: Diabetes Foot Self-Care Behavior Scale
DKS: Diabetes Knowledge Scale
ITHBC: integrated theory of health behavior change
NLEA: nontraumatic lower extremity amputation
REDCap: Research Electronic Data Capture
SDSCA: Summary of Diabetes Self-Care Activities
TUQ: Telehealth Usability Questionnaire
